# Exploring the Effects of Various Polymeric Backbones on the Performance of a Hydroxyaromatic 1,2,3-Triazole Anion Sensor

**DOI:** 10.3390/s20102973

**Published:** 2020-05-24

**Authors:** Aikohi Ugboya, Khristal Monroe, Unodinma Ofulue, Kayley Yates, Debanjana Ghosh, Shainaz M. Landge, Rafael Lopes Quirino, Karelle S. Aiken

**Affiliations:** Chemistry and Biochemistry, Georgia Southern University, Statesboro, GA 30460, USA; au00353@georgiasouthern.edu (A.U.); kd02116@georgiasouthern.edu (K.M.); uo00137@georgiasouthern.edu (U.O.); kayley.yates@hawks.huntingdon.edu (K.Y.); dghosh@georgiasouthern.edu (D.G.); slandge@georgiasouthern.edu (S.M.L.)

**Keywords:** polymeric chemosensor, 1,2,3-triazole, anion detection

## Abstract

Polymeric chemosensors are vital sensing tools because of higher sensitivity compared to their monomeric counterparts and tunable mechanical properties. This study focuses on the incorporation of a hydroxyaromatic 1,2,3-triazole sensor, 2-(4-**p**henyl 1*H*-1,2,3-**t**riazol-1-yl)**p**henol (**PTP**), into polymers. By itself, the triazole has a selective, fluorometric response to the fluoride, acetate, and dihydrogen phosphate anions, and is most responsive to fluoride. Current investigations probe the suitability of various polymeric backbones for the retention and enhancement of the triazole’s sensing capabilities. Backbones derived from acrylic acid, methyl methacrylate, divinylbenzene, and styrene were explored. UV-illumination, Nuclear Magnetic Resonance (NMR) titration, and ultraviolet-visible (UV-Vis) absorption and fluorescence spectroscopy studies are used to investigate the performance of newly synthesized polymers and the derivatives of **PTP** that serve as the polymers’ precursors. Among the polymers investigated, copolymers with styrene proved best; these systems retained the sensing capabilities and were amenable to tuning for sensitivity.

## 1. Introduction

Ions are ubiquitous, from their roles in the regulation of enzymatic behaviors, and metabolic processes to their broad use in the production of pharmaceuticals [1,2]. An imbalance or inadequacy of certain ions can have serious health consequences. With fluoride (F^−^) for example, an excess of 3–6 mg/L in drinking water causes fluorosis, and a deficiency results in tooth decay and osteoporosis [3].

Facile and cost-efficient methods for monitoring ions are very much needed [4,5,6]. Small molecular chemosensors, due to their sensitivity and simplicity, are viable detection tools for trace amounts of analytes [7,8,9]. Polymers based on such sensors have a comparative advantage over the single-molecule systems [10,11,12]. Polymeric derivatives can lead to heightened sensitivity and targeted material-design to suit the physical needs of an environment.

**Scheme 1:**
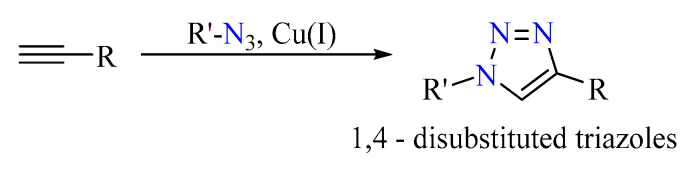
1,2,3-Triazoles via Copper-Catalyzed Azide-Alkyne Cycloaddition (CuAAC) reaction.

**Scheme 2:**
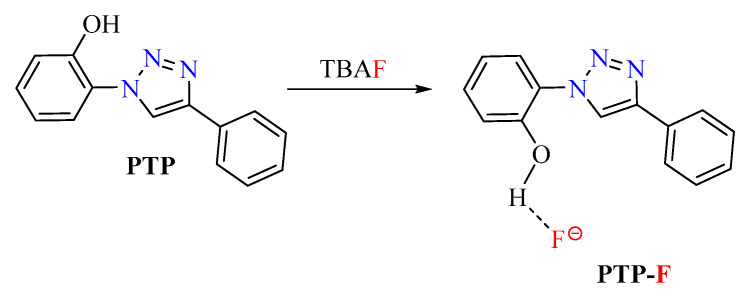
The proposed binding mode of **PTP** and fluoride.

1,2,3-Triazoles have shown excellent promise as sensors for cation and anion recognition [13,14,15]. These structures are N-donors in cation-sensing [14,16] and C*sp*^2^-H hydrogen bond donors in anion-sensing [17,18,19]. Triazoles are easily synthesized from azide and alkyne precursors via copper-catalyzed azide-alkyne cycloaddition (CuAAC) (Scheme 1) [20]. Previously, we developed an efficient 1,2,3-triazole sensor, 2-(4-**p**henyl 1*H*-1,2,3-**t**riazol-1-yl)phenol (**PTP**), that, under UV-light, ‘‘turns-on’’ in the presence of fluoride (F^−^), acetate (AcO^−^), and dihydrogen phosphate (H_2_PO_4_^−^) (Scheme 2) [21]. The response output is a blue, fluorescent signal that is strongest with fluoride. The binding constants with each of those ions are 9.0 × 10^3^ M^−1^, 6.0 × 10^3^ M^−1^, 4.9 × 10^3^ M^−1^ for F^−^, H_2_PO_4_^−^, and AcO^−^, respectively. The triazolyl ring is an integral part of the conjugative network that is responsible for the fluorescence signaling [22]. The molecule’s receptor site is the phenolic −OH with which the anion-binding cavity is created by the −OH and the triazole’s C*sp*^2^−H units (Scheme 2). During detection, OH group hydrogen bonds with the anion and the triazolyl C*sp*^2^−H via its hydrogen and oxygen, respectively [22,23]. Importantly, the turn-on signal is only elicited with anions that have the requisite basicity to deprotonate the −OH receptor site in the excited state. As such, while **PTP** detects F^−^, other halides such as bromide, chloride and iodide cannot induce a response [23]. Furthermore, as long as a signal-inducing anion can hydrogen bond to the receptor site, all evidence suggests that the turn-on response in **PTP** would not be affected by the counter ion.

**Scheme 3:**
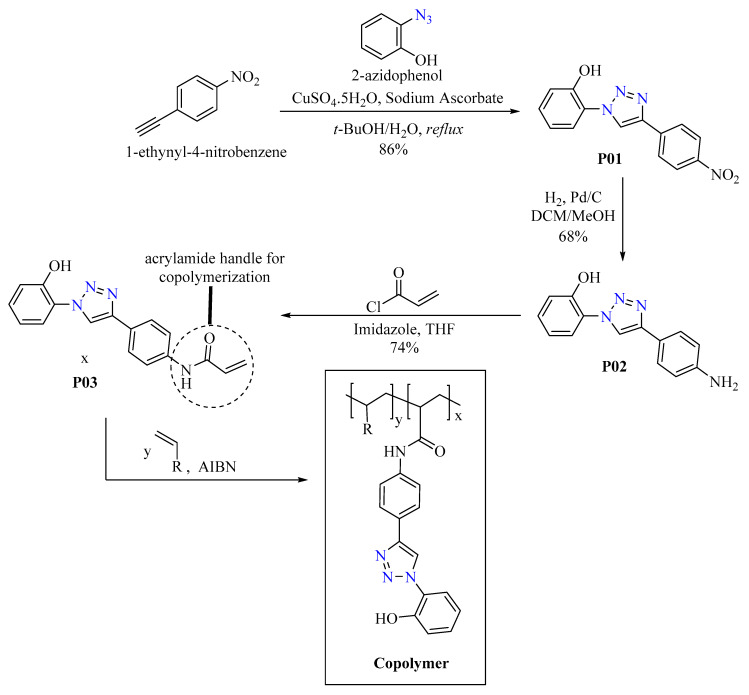
Synthesis of acrylamide monomer **P03** and its copolymers.

The work described herein consists of the immobilization of a **PTP** derivative onto a polymeric backbone. More specifically, as we look towards future applications with thin films, the main goal of the current study is to find the most suitable polymer substrates for the retention (and possible enhancement) of **PTP**’s sensing capabilities. The general approach for the polymer synthesis involves a free radical copolymerization of an acrylamide derivative of **PTP** with pre-selected traditional monomers, acrylic acid, methyl methacrylate, divinylbenzene, and styrene (Scheme 3). AIBN (azobisisobutyronitrile) is used as the free radical initiator in all cases. Copolymers that preserved the detection capabilities were subjected to detailed analyses with ultraviolet-visible (UV-Vis) and fluorescence spectroscopy. During this work, the sensing properties of **PTP**-derived polymer precursors were also assessed. This study is an important precedent for the development of tunable, supported **PTP**-based materials.

## 2. Materials and Methods

All reactants and chemicals were obtained from commercial sources and used without further purification unless noted. HPLC grade solvents and de-ionized water were used for the syntheses and all other experiments. Anhydrous Tetrahydrofuran (THF) used in reactions requiring air-free/dry conditions was obtained from MBraun Manual Solvent Purification System and stored over 4 Å molecular sieves under nitrogen gas prior to use. Flash column chromatography was performed using Sorbent technologies Silica gel with particle size 40–63 microns. Nuclear Magnetic Resonance (NMR) spectra were recorded on Agilent MR4000DD2 spectrometer with a multinuclear probe with two RF channels and variable temperature capability. ^1^H-NMR: 400 MHz and ^13^C NMR: 100 MHz; the solvents used were deuterated acetonitrile (CD_3_CN) and dimethyl sulfoxide ((CD_3_)_2_SO). NMR signals were recorded in parts per million (ppm) relative to the residual in each solvent. Signals are described as singlet (s), doublet (d), doublet of doublet (dd), triplet (t), multiplet (m); coupling constants (*J*; Hz), and with integration. Melting points were measured with Vernier Melt Station using Vernier LabQuest 2 and are uncorrected.

Room temperature absorption and steady-state fluorescence measurements were performed using a Shimadzu UV-2450 spectrophotometer and PerkinElmer LS55 with well plate reader fluorimeter, respectively. For fluorescence experiments, the scan type was emission with a single mode. The excitation wavelength was 300 nm with a scan speed of 100 nm/min and a scanning interval of 0.5 nm. The gain was medium. Scan range was 305 nm to 595 nm. The excitation slit was 5 nm and the emission slit was 2.5 nm. For absorbance experiments, the measuring mode was absorption with a wavelength range of 200 nm to 600 nm and a sampling interval of 1.0 nm. The scan mode was single, the slit width was 2.0 nm, the light source change wavelength was 360 nm, the S/R exchange was set at normal and scan speed was set as fast.

Solutions of the tetrabutylammonium salts were prepared in acetonitrile for fluorescence and UV-Vis spectroscopy investigations, and in deuterated acetonitrile for the NMR titration. If solutions of the copolymers were used, 500 mg of the polymer’s pellets were dissolved in 20 mL of the appropriate solvent (Table S1). Information regarding the solvents for the polymer solutions, equivalents, volumes, and concentrations for each experiment are provided in the figures.

### 2.1. Synthesis and Characterization of Precursors (PTP Derivatives)

2-(4-(4-nitrophenyl)-1*H*-1,2,3-triazole-1-yl)phenol (**P01**) and 2-(4-(4-Aminophenyl)-1*H*-1,2,3-triazole-1-yl)phenol (**P02**) were synthesized according the procedures in Ghosh et al. [23].

#### 2.1.1. Synthesis of 2-(4-(4-N-phenylacrylamide)-1H-1,2,3-triazole-1-yl)Phenol or P03

Compound **P02** (253 mg, 1 mmol) and imidazole (68.0 mg, 1 mmol) were dissolved in anhydrous tetrahydrofuran (10 mL) in an oven-dried, round bottom flask equipped with a stir bar and a nitrogen inlet. The mixture was cooled to at 0 °C. Acryloyl chloride (0.08 mL, 1 mmol) was added to the reaction. The reaction was stirred vigorously for 24 h. The resulting mixture was filtered through glass wool in a long stem funnel. The crude dried product was collected by rotary evaporation and purified using silica plug (50% ethyl acetate in hexanes mixed with 1.0% methanol) to give a light brown solid, 230 mg (74%). M.P.: 189.1 – 191.1 ^o^C. ^1^H NMR (400 MHz, (CD_3_)_2_SO)] δ 10.53 (s, 1-H), 10.24 (s, 1-H), 8.80 (s, 1H), 7.87 (d, *J* = 8.7 Hz, 2-H), 7.74 (d, *J* = 8.7 Hz, 2-H), 7.57 (dd, *J* = 7.9, 1.7 Hz, 1-H), 7.33 (m, 1H), 7.10 (dd, *J* = 8.3, 1.2 Hz, 1-H), 6.97 (td, *J* = 7.8, 1.3 Hz, 1-H), 6.43 (dd, *J* = 17.0, 10.1 Hz, 1-H), 6.25 (dd, *J* = 17.0, 2.0 Hz, 1-H), 5.74 (dd, *J* = 10.1, 2.0 Hz, 1-H); ^13^C-NMR [100 MHz, (CD_3_)_2_SO)] δ 163.6, 150.4, 146.3, 139.2, 132.2, 130.8, 127.5, 126.3, 126.2, 125.9, 125.0, 122.9, 120.1, 119.9, 117.4.

The structure **P03** is fully characterized by 1D and 2D NMR spectra (Supporting Information-Section II).

#### 2.1.2. Synthesis of Polymeric Chemosensors: **P04**, **P05**, **P06**, and **P07**

The incorporation of compound **P03** onto different polymer backbones was accomplished by a bulk free-radical copolymerization of **P03** with reactive co-monomers of interest, namely acrylic acid, methyl methacrylate, divinylbenzene, and styrene, generating samples **P04**, **P05**, **P06,** and **P07**, respectively. For all polymeric chemosensor syntheses, azobisisobutyronitrile (AIBN) was used as the free radical initiator. To match the concentration of chemosensor used in previous studies in solution [21,24,25], **P04**, **P05**, **P06,** and **P07** were prepared with a **P03** concentration of 0.1 mol%. Additionally, another two styrene-based chemosensors were synthesized with **P03** concentrations of 1.0 mol% and 10.0 mol%. The general procedure for the synthesis of **P04**, **P05**, **P06,** and **P07** consisted of mixing the pre-determined amounts of **P03** and 10.0 g of the desired co-monomer in a 20 mL scintillation vial. After the addition of 0.5 g of AIBN, the vial was capped and placed in a convection oven at 60 ˚C for 4 h.

## 3. Results and Discussion

### 3.1. Synthetic Approach

#### 3.1.1. Synthesis of the Monomeric Sensor

The **PTP** core was functionalized with an acrylamide group for copolymerization with other reactive unsaturated monomers (Scheme 3). The approach began with the synthesis of nitro analog **P01** using the CuAAC reaction between 2-azidophenol and 1-ethynyl-4-nitrobenzene. **P01** was subsequently reduced to **P02** in which the inclusion of the amino group served two purposes. First, the amino group was reacted with acryloyl chloride to provide the desired monomer **P03**. Second, the electron-donating ability of the amino group enhances the fluorescent response of the sensor [23,26,27,28]. **P03** was synthesized from **P02** in 74% yield.

#### 3.1.2. Synthesis of Polymers

**Figure 1:**
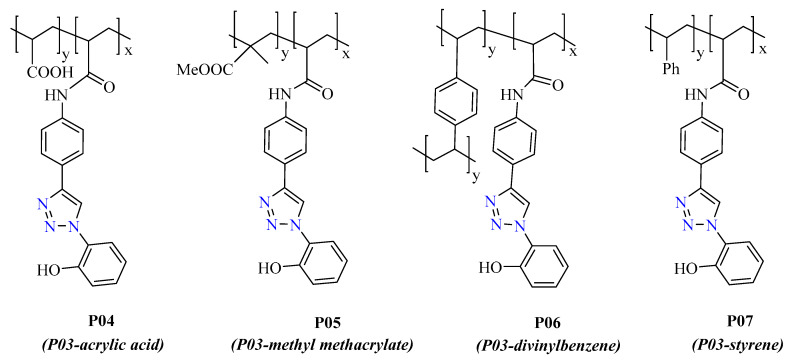
Structures of **PTP**-based polymeric chemosensors.

The general synthetic route for the copolymerization with **P03** is shown in Scheme 3. **P03** was reacted with acrylic acid, methyl methacrylate, divinylbenzene, and styrene to make **P04**, **P05**, **P06,** and **P07**, respectively (Figure 1). In all cases, AIBN was used as the initiator for bulk free-radical polymerization. The polymeric sensors prepared with acrylic acid, methyl methacrylate, and divinylbenzene were made with a **P03** concentration of 0.1 mol% to mimic the solution concentration of **PTP** used in earlier sensing studies [21,24,25]. This allowed for a comparative analysis between earlier data and the immobilized sensors described here. With styrene, samples containing 1.0 mol% and 10.0 mol% **P03** were also prepared.

### 3.2. Anion Detection

The response of the polymeric sensors and their precursors to the tetrabutylammonium (TBA) salts of different anions were investigated. Studies under visible and ultraviolet (UV) light were used to determine if the unique detection properties of the **PTP** core were retained at each step. These studies probed whether or not F^−^, AcO^−^, and H_2_PO_4_^−^ still induced a selective, blue, fluorescent response for which the strongest signal output occurs with F^−^.

With the exception of **P06**, all investigations were carried out in acetonitrile or co-solvent mixtures based on each copolymer’s solubility (Table S1). **P06** was insoluble in all of the solvents tested.

#### 3.2.1. Response with Synthetic Precursors

Screening studies with **P01/2/3** were carried out in acetonitrile with TBA salts (ClO_4_^−^, F^−^, Br^−^, H_2_PO_4_^−^, I^−^, Cl^−^, AcO^−^, BF_4_^−^) at a molar ratio of 1:1 (**P01/2/3**: salt) under UV (365 nm) and ambient light.

**Figure 2:**
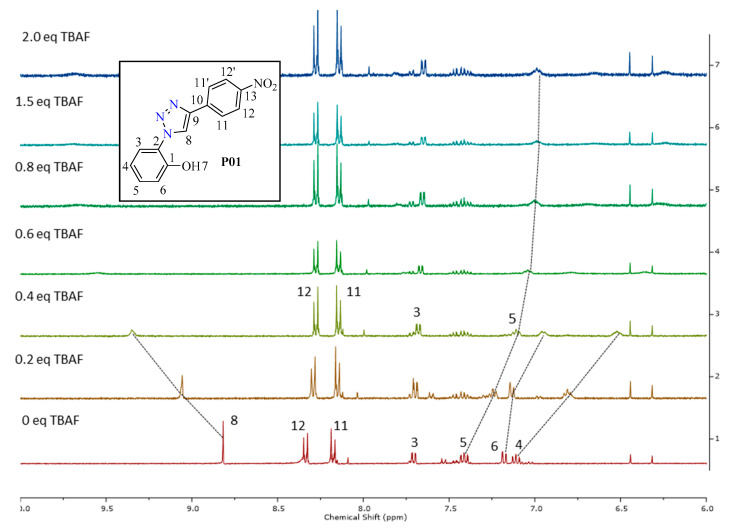
^1^H NMR experiments with **P01** and increasing equivalents of TBAF in deuterated acetonitrile (CD_3_CN).

Under ambient and UV light **P01** was non-responsive to all the anions including H_2_PO_4_^−^, AcO^−^, and F^−^ (Figure S1). To verify that the non-response under UV illumination was due to the nitro group and not a compromised anion-receptor site, an NMR titration experiment with **P01** and TBAF was performed (Figure 2). With increasing equivalents of TBAF, a downfield shift of the triazole proton (H-8) and an upfield shift of the phenol’s H-5, H-6, and H-4 occurred. Eventually, the resonances of H-8, H-6, and H-4 broadened out into the baseline of the spectra. Consistent with the behavior of **PTP** and similar molecules [14,21,24,25], these changes in the H-8 and phenol group’s chemical shifts indicated coordination between **P01′**s −OH unit and F^−^ (Scheme 2 and Figure 2). Thus, the non-fluorometric response of **P01** was solely due to the quenching effect of the nitro group [29,30,31]. The anion-receptor site was intact.

Once the nitro group was converted to −NH_2_, the turn-on, blue fluorescent signal for anion detection was recovered with **P02 [23]**. Screening with the TBA salts showed a selective response to H_2_PO_4_^−^, AcO^−^, and F^−^where the intensity of signal-output was greatest with F^−^and weakest with H_2_PO_4_^−^. Similar results were obtained with the acrylamide **P03** which gave a distinct signal-output for F^−^ and AcO^−^ (Figure S2).

#### 3.2.2. Response with Copolymers **P04**, **P05**, **P06**, and **P07**

The anion detection abilities of copolymers with acrylic acid **P04**, methyl methacrylate **P05**, divinylbenzene **P06**, and styrene **P07** were explored. Promising results were obtained with **P07**. With the exception of **P06**, the sensing behavior of each polymer was studied in an aprotic solvent in which the polymer was most soluble (Table S1). It should be noted that the choice of solvent for the studies below is based on polymers’ solubility and the absence of H-bond donors in the solvent molecules. H-bond donors in the solvent would compete with the sensor unit’s −OH receptor site.

**Figure 3:**
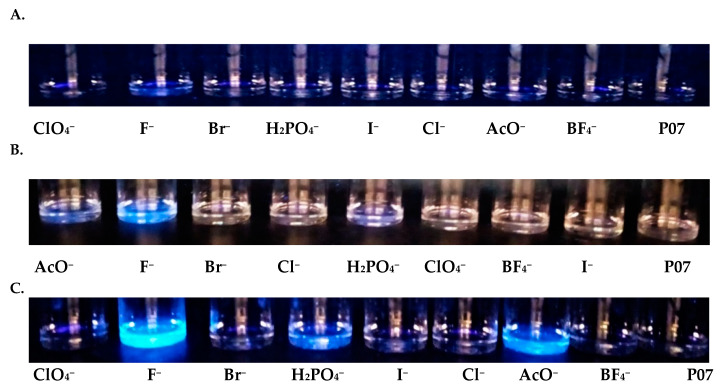
Response of **P07** (solvent: toluene) with **P03** loadings of (**A**) 0.1 mol%, (**B**) 1.0 mol%, and (**C**) 10.0 mol% to TBA salts (Concentration of stock solution: 1.98 × 10^−3^ M, solvent: acetonitrile) under UV light (365 nm). Volume of anion solution: Volume of **P07** solution, 1:1.

With **P07** (**P03**-styrene, 0.1 mol% **P03**) in toluene solution, initial investigation under UV light showed an exclusive, blue fluorescent response to F^-^ at the anion concentrations studied (Figure 3). Higher **P03** loading of 1.0 mol% and 10.0 mol% increased the intensity of that response and in the process, yielded polymers that also detected H_2_PO_4_^−^ and AcO^−^. As with the native molecule **PTP**, under ambient light, the appearance of the **P07** solutions did not change regardless of the anion present (Figure S6). Overall, the variation in detection performance with the different sensor loadings points to the capacity to tune the sensitivity of **P07**-type chemosensors using the **P03** mol% content.

The side chains of **P04-6** proved incompatible with the **PTP** core’s sensing mechanism. **P04** (**P03-** acrylic acid) in DMF solution fluoresced blue in the absence and presence of all salts under the 365nm lamp (Figure S3). This polymer has H-bond donating −COOH side chains that are most likely disrupting the phenolic −OH-anion interaction needed for a signal-response [22,23]. **P05** (**P03**-methyl methacrylate) in chloroform is non-fluorescent under UV light and remains that way when treated with the anions (Figure S4). In this case, the poly(methyl methacrylate) backbone may be quenching fluorescence, possibly through a process like photoinduced electron transfer (PET) [32,33,34]. **P06** (**P03**-divinylbenzene) was insoluble in all of the solvents tested and as such, screening was performed with solid samples. **P06** with its cross-linked aryl side chains fluoresces under UV illumination (Figure S5) [35,36,37]. Treatment with drops of the anion solutions on **P06** pellets failed to elicit any type of signal.

#### 3.2.3. Absorbance Spectroscopy

The absorbance studies focused on the behavior of the **P07** (0.1, 1.0, and 10.0 mol% **P03**) with F^−^ The λ*_max_* before and after the addition of fluoride was the same, 292 nm for all **P03** loadings (Figure S7). The 292 nm band is due to the polystyrene backbone [38]. Treatment with TBAF produced slight variations in the λ*_max_* intensities.

#### 3.2.4. Fluorescence Spectroscopy

**Figure 4:**
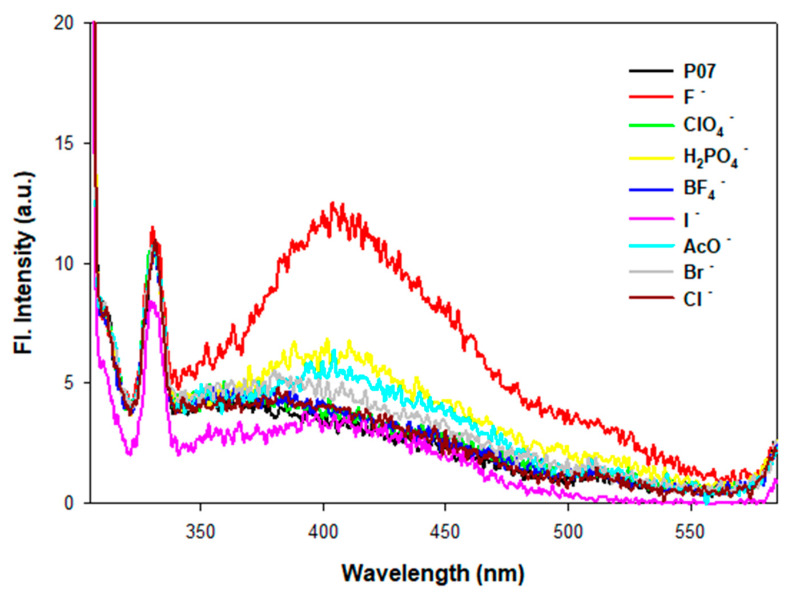
Fluorescence spectra of **P07** (0.1 mol% **P03**, solvent: toluene) treated with TBA salts (3.88 × 10^−5^ M, stock solution solvent: acetonitrile). Excitation at 300 nm. Volume of **P07** solution = 2.5 mL.

**P07** (0.1 mol% **P03**) was used to explore the polymer’s response to a series of anions (ClO_4_^−^, F^−^, Br^−^, H_2_PO_4_^−^, I^−^, Cl^−^, AcO^−^, BF_4_^−^) (Figure 4). An excitation wavelength of 300 nm was used for these studies. The excitation wavelengths for the parent **PTP** and the solvent, toluene, are within a few nanometers of each other [21,39]. **PTP**’s excitation wavelength was determined to be 290 nm. As such, a slightly longer excitation wavelength of 300 nm was used in the current work so that a credible comparison between **P07** and **PTP** could be made while also preventing interference from the solvent. Consistent with the visual observations in Figure 3 and the behavior of **PTP** (with F^–^, fluorescence λ*_max_*= 430 nm), the most distinct change occurred with the addition of TBAF. The polymer had a weak fluorescence λ*_max_* at ~395 nm which, in the presence of fluoride, red-shifted to 410 nm. The latter signal registered a considerable increase in the intensity as well.

**Figure 5:**
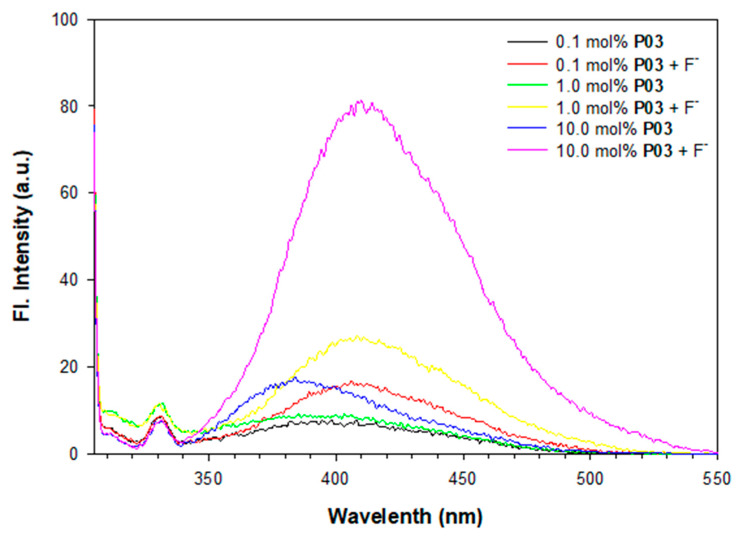
Fluorescence spectra of **P07** (0.1, 1.0, and 10.0 mol% **P03**, solvent: toluene) with TBAF (3.88 × 10^−5^ M, stock solution solvent: acetonitrile). Excitation at 300 nm. Volume of **P07** solution = 2.5 mL.

The F^−^ detection capabilities of **P07** with three loadings of **P03** were investigated (Figure 5). The **P07** fluorescence λ*_max_* was the same for 0.1 and 1.0 mol% **P03**, ~395 nm, but different for 10 mol% **P03**, 385 nm. In all cases, the addition of TBAF resulted in a bathochromic shift to 410 nm and a marked increase in intensity. Importantly, the signal intensity during detection was proportional to the **P03** content.

**Figure 6:**
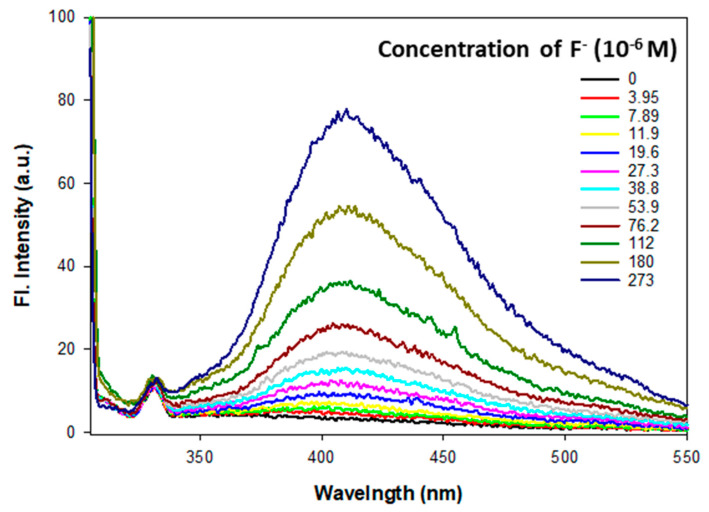
Fluorescence spectra of **P07** (0.1 mol% **P03**, solvent: toluene) treated with TBAF (stock solution solvent: acetonitrile). Excitation at 300 nm. Volume of **P07** solution = 2.5 mL.

**Figure 7:**
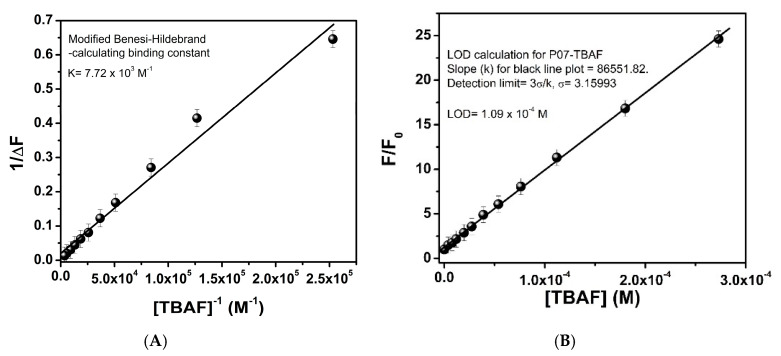
Analysis of the **P07**-fluoride interaction with (**A**) a Benesi–Hildebrand plot and (**B**) a calibration curve.

Fluorescence titration studies probed the behavior of **P07** (0.1 mol% **P03**) with varying concentrations of fluoride (Figure 6). As anticipated, the 410 nm detection signal became stronger with increasing equivalents of fluoride. A Benesi–Hildebrand plot of the inverse of the change in fluorescence intensity (1/ΔF) versus the inverse of fluoride concentration [TBAF]^−1^ was linear suggesting a 1:1 binding interaction between the **P07′**s sensor unit and fluoride (Figure 7A). Based on this plot, the binding constant (K) is 7.72 × 10^3^ M^−1^. The stoichiometry and K for the **P07**-fluoride interaction are similar to that of the parent **PTP** for which the ratio is 1:1, **PTP**:fluoride, and K= 9 × 10^3^ M^−1^ [21].

The following equation was used to calculate K [40]:

1/ΔF = 1/ΔF*_max_* + 1/K ∙ 1/ΔF*_max_* ∙ 1/[TBAF] (1)

where ΔF = F*_x_* – F_0_; ΔF*_max_* = F_∞_ – F_0_. F_0_, F*_x_* and F_∞_ are the fluorescence intensities of **P07** in the absence, at an intermediate concentration, and a concentration of complete interaction with the anion.

Significantly, **P07′s** limit of detection (LOD), ~1.09 × 10^−4^ M, is below the recommended level of fluoride in drinking water (3–6 mg/L) (Figure 7B) [3]. The LOD was determined with a calibration curve which is also linear, a plot of F/F_0_ versus [TBAF] where F is the fluorescence intensity after the addition of the anion.

## 4. Conclusions

The suitability of polymeric backbones for the immobilization of an anion sensor (**PTP**) and the retention of its detection capabilities were explored using copolymers derived from an acrylamide derivative of **PTP** and various unsaturated monomers (acrylic acid, methyl methacrylate, divinylbenzene, and styrene). The parent molecule **PTP** selectively detects fluoride (F^−^), acetate (AcO^−^), and dihydrogen phosphate (H_2_PO_4_^−^) with a blue, turn-on fluorescent response. Copolymerization with styrene (0.1, 1.0 and 10.0 mol% **PTP**-acrylamide), **P07**, preserved the sensing properties; all other backbones muted the detection signal. Furthermore, studies using UV-illumination and fluorescence spectroscopy demonstrated that the sensitivity of **P07** could be controlled with the sensor loading. At the anion concentrations used in these studies, an exclusive response to F^-^ was obtained with the copolymer comprised of 0.1 mol% sensor. Higher loadings of 1.0 mol% and 10.0 mol%, gave more intense signals with F^-^ as well as responses to AcO^−^ and H_2_PO_4_^−^. The performance of the **PTP**-derived precursors for the copolymers were also studied. With the exception of the nitro derivative **P01**, the detection abilities of the precursors **P02** and **P03** were consistent with the parent molecule. Overall, the results of this investigation show great promise for the development of tunable, supported, **PTP**-based materials copolymerized with styrene. Future work will involve the investigation of copolymer **P07** as a solid device (thin film and coated paper).

## Supplementary Materials

Available online at https://www.mdpi.com/1424-8220/20/10/2973/s1. Nuclear Magnetic Resonance Spectra: Compound **P03,** ^1^H-NMR Spectrum [Solvent: (CD_3_)_2_SO]; Compound **P03,** ^13^C-NMR Spectrum [Solvent: (CD_3_)_2_SO]; Compound **P03,** 2D COSY Spectrum [Solvent: (CD_3_)_2_SO]; Compound **P03,** 2D HSQC Spectrum [Solvent: (CD_3_)_2_SO]. Solubility Test: Table S1. Solubility Test for Polymers. Figure S1. Response of **P01** (Concentration of stock solution: 1.98 × 10^−3^M, solvent: acetonitrile) treated with of different TBA salts of anions (Concentration of stock solution: 1.98 × 10^−3^M, solvent: acetonitrile) under (A) ambient and (B) ultraviolet light (365 nm). Volume of anion solution: Volume of **P01** solution, 1:1. Figure S2. Response of **P03** (Concentration of stock solution: 1.98 × 10^−3^ M, solvent: acetonitrile) treated with of different TBA salts of anions (Concentration of stock solution: 1.98 × 10^−3^ M, solvent: acetonitrile) under (A) ambient and (B) ultraviolet light (365 nm). Volume of anion solution: Volume of **P03** solution, 1:1. Figure S3. **P04** (solvent: DMF) treated with different TBA salts of anions (Concentration of stock solution: 1.98 × 10^−3^ M, solvent: acetonitrile) under (A) ambient and (B) ultraviolet light (365 nm). Volume of anion solution: Volume of **P04** solution, 1:1. Figure S4. **P05** (solvent: chloroform) treated with different TBA salts of anions (Concentration of stock solution: 1.98 × 10^−3^ M, solvent: acetonitrile) under (A) ambient and (B) ultraviolet light (365 nm). Volume of anion solution: Volume of **P05** solution, 1:1. Figure S5. **P06** treated with different TBA salts of anions (Concentration of stock solution: 1.98 x 10^−3^ M, solvent: acetonitrile) under (A) ambient and (B) ultraviolet light (365 nm). Volume of anion solution= 1.0 mL. Figure S6. Response of **P07** (solvent: toluene) with **P03** loadings of (A) 0.1 mol%, (B) 1.0 mol%, and (C) 10.0 mol% to TBA salts (Concentration of stock solution: 1.98 × 10^−3^ M, solvent: acetonitrile) under ambient light. Volume of anion solution: Volume of **P07** solution, 1:1. Figure S7. Absorbance spectrum of **P07** (solvent: toluene) with TBAF (3.88 × 10^−5^ M, stock solution solvent: acetonitrile). Volume of **P07** solution = 2.5 mL.
