# Supplementary material for: Exploring the Effects of Various Polymeric Backbones on the Performance of a Hydroxyaromatic 1,2,3-Triazole Anion Sensor
Source: Sensors (Basel). 2020 May 24;20(10):2973. doi: 10.3390/s20102973 (PMC7287593; doi:10.3390/s20102973)
Supplement: Supplementary file 1 [file sensors-20-02973-s001.pdf]

Exploring the effects of various polymeric backbones on the performance of a hydroxyaromatic 1,2,3-triazole anion sensor

Authors: Aikohi Ugboya [1], Khristal Monroe [1], Unodinma Ofulue[1], Kayley Yates[1], Debanjana Ghosh [1], Shainaz M. Landge [1], Rafael Lopes Quirino[1,*], and Karelle S. Aiken [1,*]

[1.]Chemistry & Biochemistry, Georgia Southern University, Statesboro, GA 30460
*Correspondence: rquirino@georgiasouthern.edu, kaiken@georgiasouthern.edu

Supporting Information

Table of Contents

I. General Experimental S2
II. Nuclear Magnetic Resonance Spectra S3-4
III. Solubility Tests S5
IV. Detection Studies under Ambient and Ultraviolet (UV) Light S6-8
V. Absorbance Studies with **P07** S9

I. General Experimental: All reactants and chemicals were obtained from commercial sources and used without further purification unless noted. HPLC grade solvents and de-ionized water were used for the syntheses and all other experiments. Anhydrous Tetrahydrofuran (THF) used in reactions requiring air-free/dry conditions was obtained from MBraun Manual Solvent Purification System and stored over 4 Å molecular sieves under nitrogen gas prior to use. Flash column chromatography was performed using Sorbent technologies Silica gel with particle size 40 – 63 microns. Nuclear Magnetic Resonance (NMR) spectra were recorded on Agilent MR4000DD2 spectrometer with a multinuclear probe with two RF channels and variable temperature capability. ^{1}H-NMR: 400 MHz and ^{13}C NMR: 100 MHz; the solvents used were deuterated acetonitrile (CD_3CN) and dimethyl sulfoxide ($(CD_3)_2SO$). NMR signals were recorded in parts per million (ppm) relative to the residual in each solvent. Signals are described as: singlet (s), doublet (d), doublet of doublet (dd), triplet (t), multiplet (m); coupling constants (*J*; Hz) and with integration. Melting points were measured with Vernier Melt Station using Vernier LabQuest 2 and are uncorrected.

Room temperature absorption and steady state fluorescence measurements were performed using a Shimadzu UV-2450 spectrophotometer and PerkinElmer LS55 with well plate reader fluorimeter, respectively. For fluorescence experiments, the scan type was emission with single mode. The excitation wavelength was 300 nm with a scan speed of 100 nm/min and a scanning interval of 0.5 nm. The gain was medium. Scan range was 305 nm to 595 nm. The excitation slit was 5 nm and the emission slit was 2.5 nm. For absorbance experiments, the measuring mode was absorption with a wavelength range of 200 nm to 600 nm and a sampling interval of 1.0 nm. The scan mode was single, the slit width was 2.0 nm, the light source change wavelength was 360 nm, the S/R exchange was set at normal and scan speed was set as fast.

Solutions of the tetrabutylammonium salts were prepared in acetonitrile for fluorescence and UV-Vis spectroscopy investigations, and in deuterated acetonitrile for the NMR titration. If solutions of the copolymers were used, 500 mg of the polymer's pellets were dissolved in 20 mL of the appropriate solvent (Table S1). Information regarding the solvents for the polymer solutions, equivalents, volumes, and concentrations for each experiment are provided in the figures.

II. Nuclear Magnetic Resonance Spectra

Compound **P03,** ^{1}H-NMR Spectrum [Solvent: $(CD_3)_2SO$]

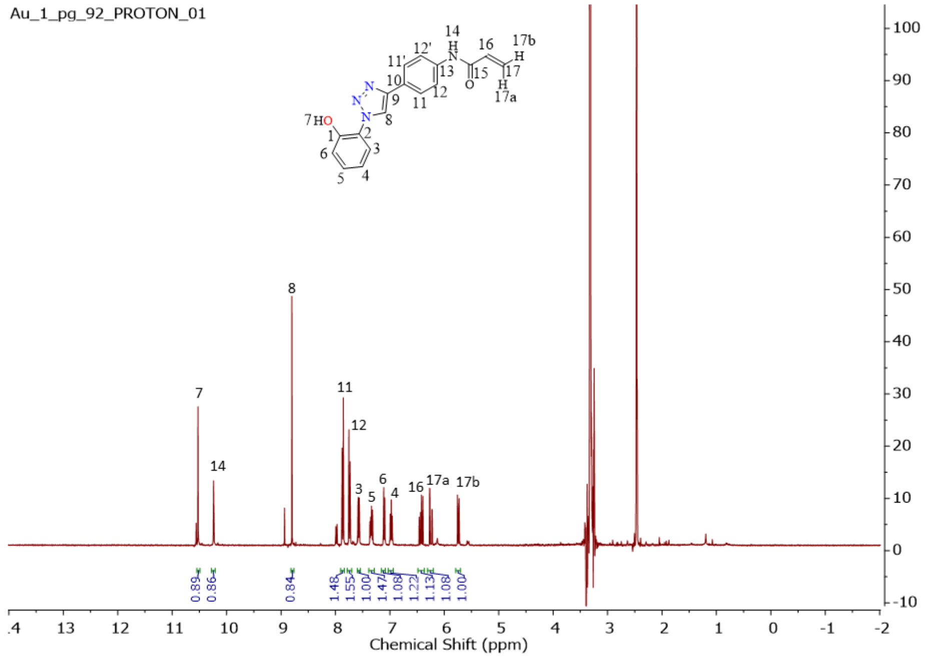

Compound **P03,** ^{13}C-NMR Spectrum [Solvent: $(CD_3)_2SO$]

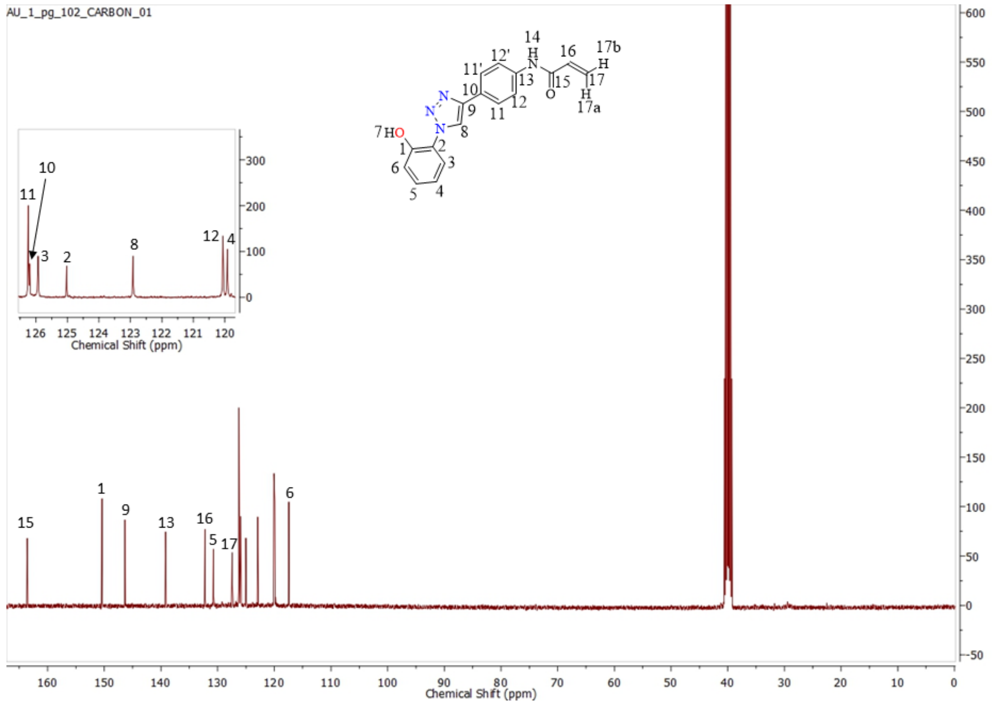

Compound **P03,** 2D COSY Spectrum [Solvent: $(CD_3)_2SO$]

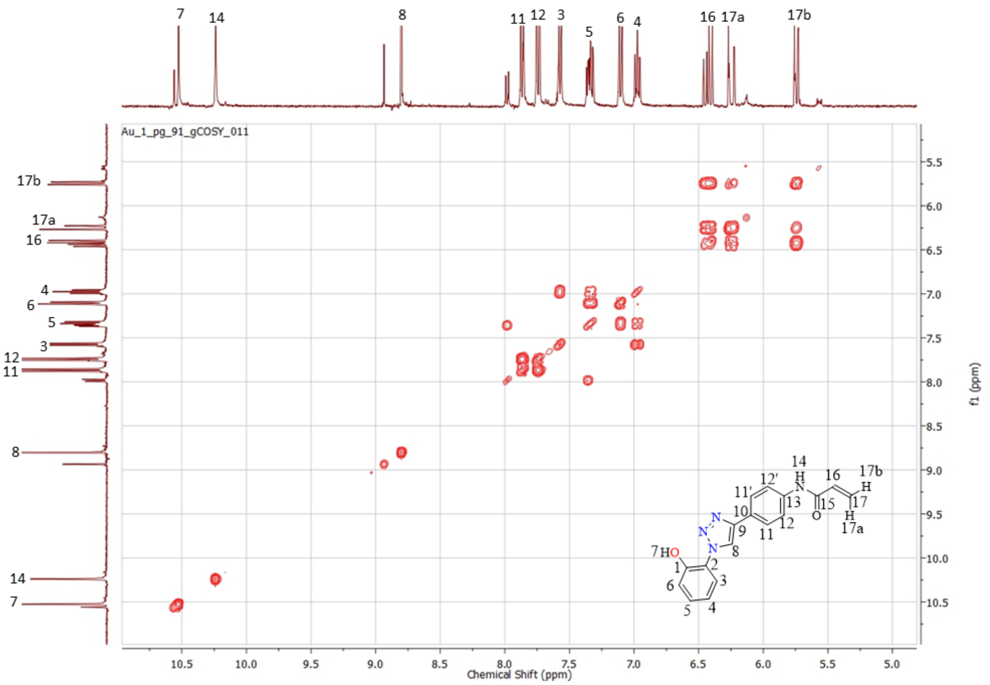

Compound **P03,** 2D HSQC Spectrum [Solvent: $(CD_3)_2SO$]

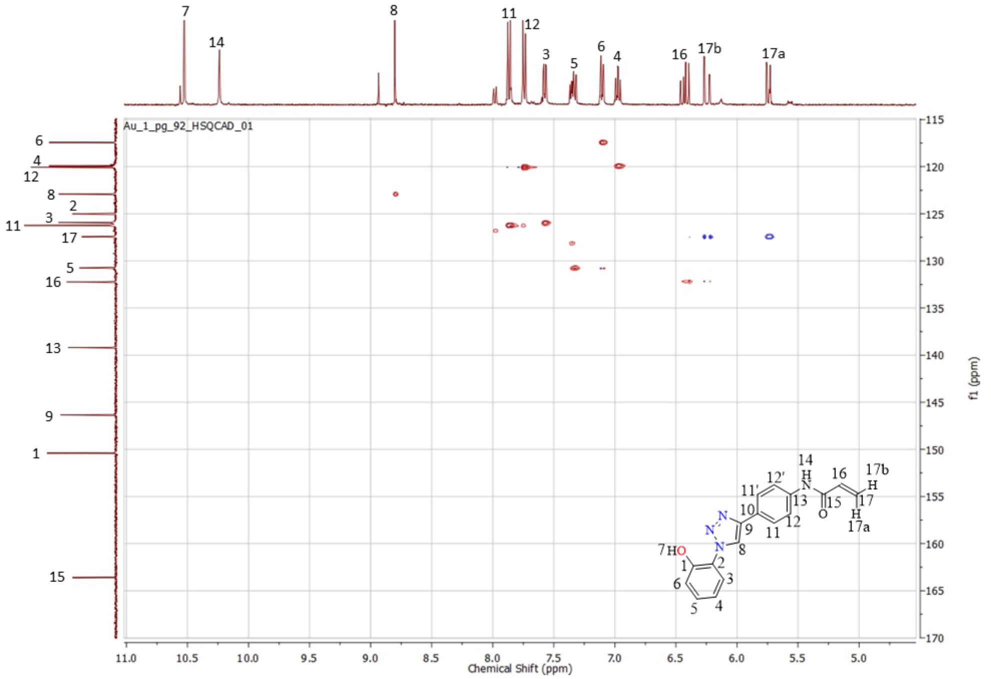

III. Solubility Test

Table S1. Solubility Test for Polymers

Solvent	Copolymer[a]			
	**P04**	**P05**	**P06**	**P07**
Hexanes	IS	IS	IS	IS
Ethanol	SO	IS	IS	IS
Methanol	SO	IS	IS	IS
Dimethyl Formamide	SO	SS	IS	SS
Methylene Chloride	IS	SO	IS	SO
Ethyl Acetate	IS	SO	IS	SO
Acetonitrile	IS	SO	IS	IS
Chloroform	IS	SO	IS	SO
Toluene	IS	IS	IS	SO

a) SO- Soluble, SS- Sparingly soluble, IS- Insoluble

IV. Detection Studies under Ambient and Ultraviolet (UV) Light

A. Ambient Light

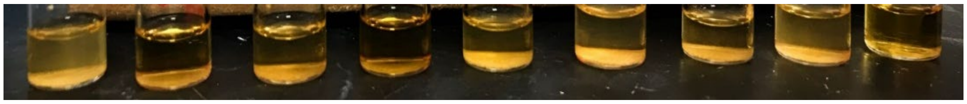

ClO_4^- F^- Br^- $H_2PO_4^-$ I^- Cl^- AcO^- BF_4^- P01

B. Ultraviolet Light (365nm)

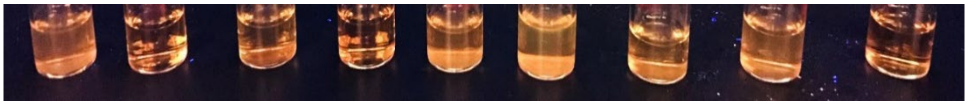

ClO_4^- F^- Br^- $H_2PO_4^-$ I^- Cl^- AcO^- BF_4^- P01

Figure S1. Response of **P01** (Concentration of stock solution: 1.98 x 10^{-3}M, solvent: acetonitrile) treated with of different TBA salts of anions (Concentration of stock solution: 1.98 x 10^{-3}M, solvent: acetonitrile) under (A) ambient and (B) ultraviolet light (365 nm). Volume of anion solution:Volume of **P01** solution, 1:1.

A. Ambient Light

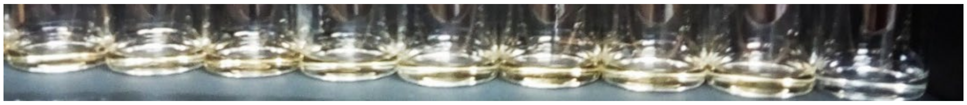

ClO_4^- F^- Br^- $H_2PO_4^-$ I^- Cl^- AcO^- BF_4^- P03

B. Ultraviolet Light (365nm)

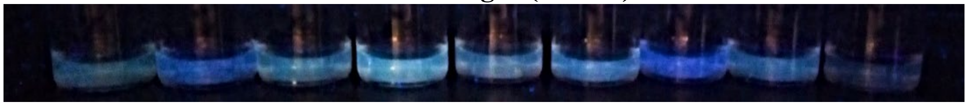

ClO_4^- F^- Br^- $H_2PO_4^-$ I^- Cl^- AcO^- BF_4^- P03

Figure S2. Response of **P03** (Concentration of stock solution: 1.98 x 10^{-3} M, solvent: acetonitrile) treated with of different TBA salts of anions (Concentration of stock solution: 1.98 x 10^{-3} M, solvent: acetonitrile) under (A) ambient and (B) ultraviolet light (365 nm). Volume of anion solution:Volume of **P03** solution, 1:1.

A. Ambient Light

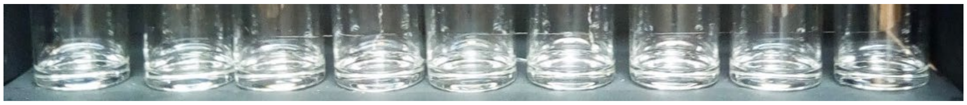

ClO_4^- F^- Br^- $H_2PO_4^-$ I^- Cl^- AcO^- BF_4^- P04

B. Ultraviolet Light (365nm)

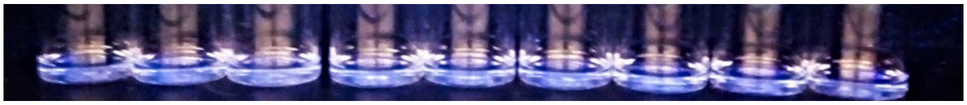

ClO_4^- F^- Br^- $H_2PO_4^-$ I^- Cl^- AcO^- BF_4^- P04

Figure S3. P04 (solvent: DMF) treated with different TBA salts of anions (Concentration of stock solution: 1.98 x 10^{-3} M, solvent: acetonitrile) under (A) ambient and (B) ultraviolet light (365 nm). Volume of anion solution:Volume of **P04** solution, 1:1.

A. Ambient Light

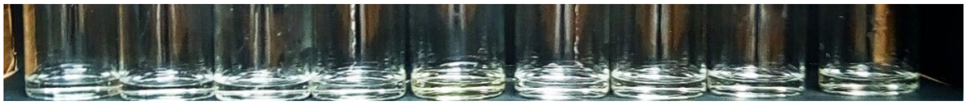

ClO_4^- F^- Br^- $H_2PO_4^-$ I^- Cl^- AcO^- BF_4^- P05

B. Ultraviolet Light (365nm)

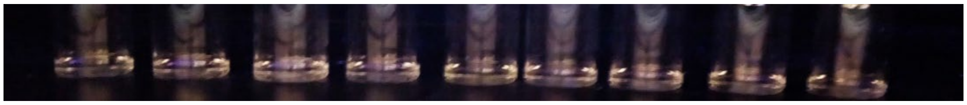

ClO_4^- F^- Br^- $H_2PO_4^-$ I^- Cl^- AcO^- BF_4^- P05

Figure S4. P05 (solvent: chloroform) treated with different TBA salts of anions (Concentration of stock solution: 1.98 x 10^{-3} M, solvent: acetonitrile) under (A) ambient and (B) ultraviolet light (365 nm). Volume of anion solution:Volume of **P05** solution, 1:1.

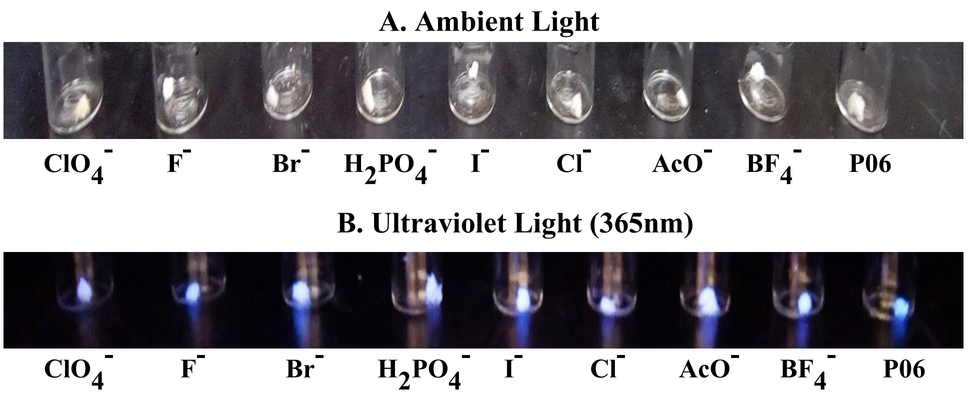

Figure S5. P06 treated with different TBA salts of anions (Concentration of stock solution: 1.98 x 10^{-3} M, solvent: acetonitrile) under (A) ambient and (B) ultraviolet light (365 nm). Volume of anion solution = 1.0 mL.

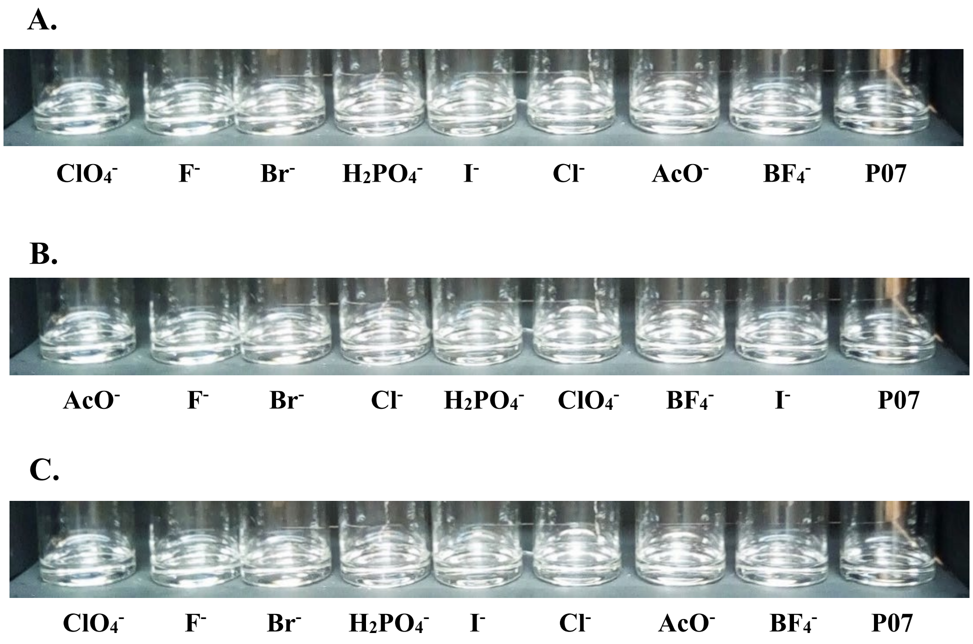

Figure S6. Response of **P07** (solvent: toluene) with **P03** loadings of (A) 0.1 mol%, (B) 1.0 mol%, and (C) 10.0 mol% to TBA salts (Concentration of stock solution: 1.98 × 10^{-3} M, solvent: acetonitrile) under ambient light. Volume of anion solution:Volume of **P07** solution, 1:1.

V. Absorbance Studies with P07

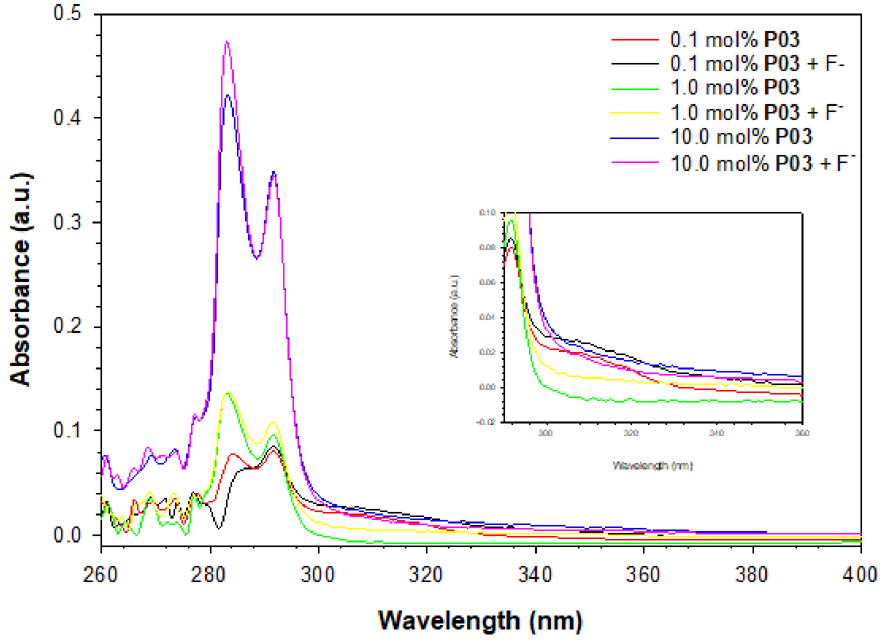

Figure S7. Absorbance spectrum of **P07** (solvent: toluene) with TBAF (3.88×10^{-5} M, stock solution solvent: acetonitrile). Volume of **P07** solution = 2.5 mL.
